# Advances in Targeting Central Cholinergic Dysfunction for Neurodegenerative Diseases: From Pharmacotherapy to Neuromodulation

**DOI:** 10.1002/cns.71151

**Published:** 2026-09-11

**Authors:** Lei Lei, Xiaoyu Hu, Ruida Lu, Jian‐jun Yang, Qingsheng Meng

**Affiliations:** ^1^ Department of Anesthesiology, Pain and Perioperative Medicine The First Affiliated Hospital of Zhengzhou University Zhengzhou China; ^2^ Department of Thyroid Surgery The First Affiliated Hospital of Zhengzhou University Zhengzhou China; ^3^ Department of Anesthesiology, Jinling Hospital, Affiliated Hospital of Medical School Nanjing University Nanjing China; ^4^ Department of Anesthesiology and Perioperative Medicine Henan Provincial People's Hospital, People's Hospital of Zhengzhou University Zhengzhou China

**Keywords:** acetylcholinesterase inhibitors, central cholinergic system, neurodegenerative diseases, neuromodulation

## Abstract

**Background:**

The central cholinergic system has long been a cornerstone therapeutic target for neurodegenerative diseases, including Alzheimer's disease (AD), Parkinson's disease dementia (PDD), and dementia with Lewy bodies (DLB). For decades, acetylcholinesterase inhibitors (AChEIs) have served as the standard symptomatic treatment, providing cognitive and functional relief by enhancing synaptic acetylcholine levels. However, their limited efficacy and inability to modify disease progression underscore the fundamental constraint of purely neurochemical enhancement, especially in the context of progressive cholinergic neuron loss.

**Results and Conclusion:**

This review critically examines the evolution of cholinergic therapies beyond AChEIs. We first explore the shift from broad neurotransmitter enhancement toward precision targeting of receptor subtypes and the development of multi‐target pharmacological strategies. Furthermore, we highlight how neuromodulation techniques—including vagus nerve stimulation, deep brain stimulation, and non‐invasive brain stimulation—directly engage and restore dysfunctional neural circuits, moving beyond mere chemical enhancement. Emerging directions such as advanced cholinergic imaging, gene therapy, and cell‐based regeneration are also discussed as promising pathways toward true disease modification. Ultimately, the integration of sophisticated pharmacological agents with circuit‐level neuromodulation represents the next frontier in treating cholinergic dysfunction across the spectrum of neurodegenerative disorders, advancing the therapeutic goal from chemical enhancement to circuit repair and regeneration.

## Graphical abstract

This review charts the evolution of cholinergic therapies for neurodegenerative diseases—from classical acetylcholinesterase inhibitors (AChEIs) providing symptomatic relief to precision multi‐target drugs and neuromodulation (VNS, DBS, NIBS) that restore dysfunctional circuits. Emerging gene therapy, cell regeneration, and advanced cholinergic imaging are also highlighted. The framework shifts the therapeutic goal from neurochemical enhancement to circuit repair and regeneration.

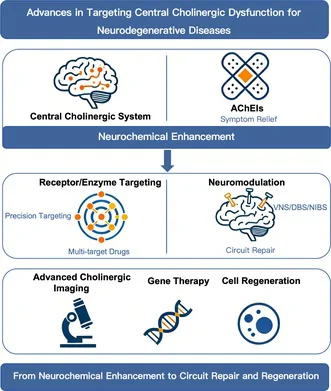

## 1 Introduction

Since the introduction of the cholinergic hypothesis, the central cholinergic system has been regarded as a core therapeutic target for neurodegenerative diseases [1, 2, 3]. In addition to Alzheimer's disease (AD), Parkinson's disease (PD), dementia with Lewy bodies (DLB), and other diseases also exhibit varying degrees of cholinergic denervation [4, 5]. This functional impairment extends beyond memory and attention, affecting a broad range of physiological functions, including gait control, emotional regulation, and sleep–wake cycles [6, 7, 8]. For decades, acetylcholinesterase inhibitors (AChEIs) have been the standard symptomatic treatment to alleviate symptoms in patients with AD and similar conditions by increasing acetylcholine (ACh) levels in the synaptic cleft [9, 10, 11]. However, the efficacy of AChEIs remains limited, with no effect on disease progression, highlighting the fundamental inadequacy of the neurochemical enhancement approach. In the context of the gradual loss of cholinergic neurons, simply increasing global ACh concentration is insufficient to precisely repair the damaged neural circuits [12, 13, 14].

At the same time, the rapid development of molecular imaging and network‐based neuromodulation techniques has enabled the in vivo visualization of the spatiotemporal dynamics of the cholinergic system and the possibility of intervening at the level of neural circuits rather than just neurotransmitter levels [15, 16]. This progress has led to the integration of pharmacological treatments with neuromodulation strategies, creating a new model of comprehensive therapy. Through these technologies, we are not only gaining a deeper understanding of the disease mechanisms but also advancing therapeutic strategies from traditional neurochemical enhancement to more precise and systematic restorative approaches.

This review will explore pharmacological advances that are already in or nearing clinical translation, followed by emerging neuromodulation strategies with growing clinical evidence. We also provide a forward‐looking perspective on enabling technologies—including advanced imaging biomarkers and gene/cell‐based approaches—where the primary value lies in highlighting future directions rather than providing exhaustive clinical validation.

## 2 Pharmacological Advances: From Single‐Target to Multi‐Target Approaches

**FIGURE 1:**
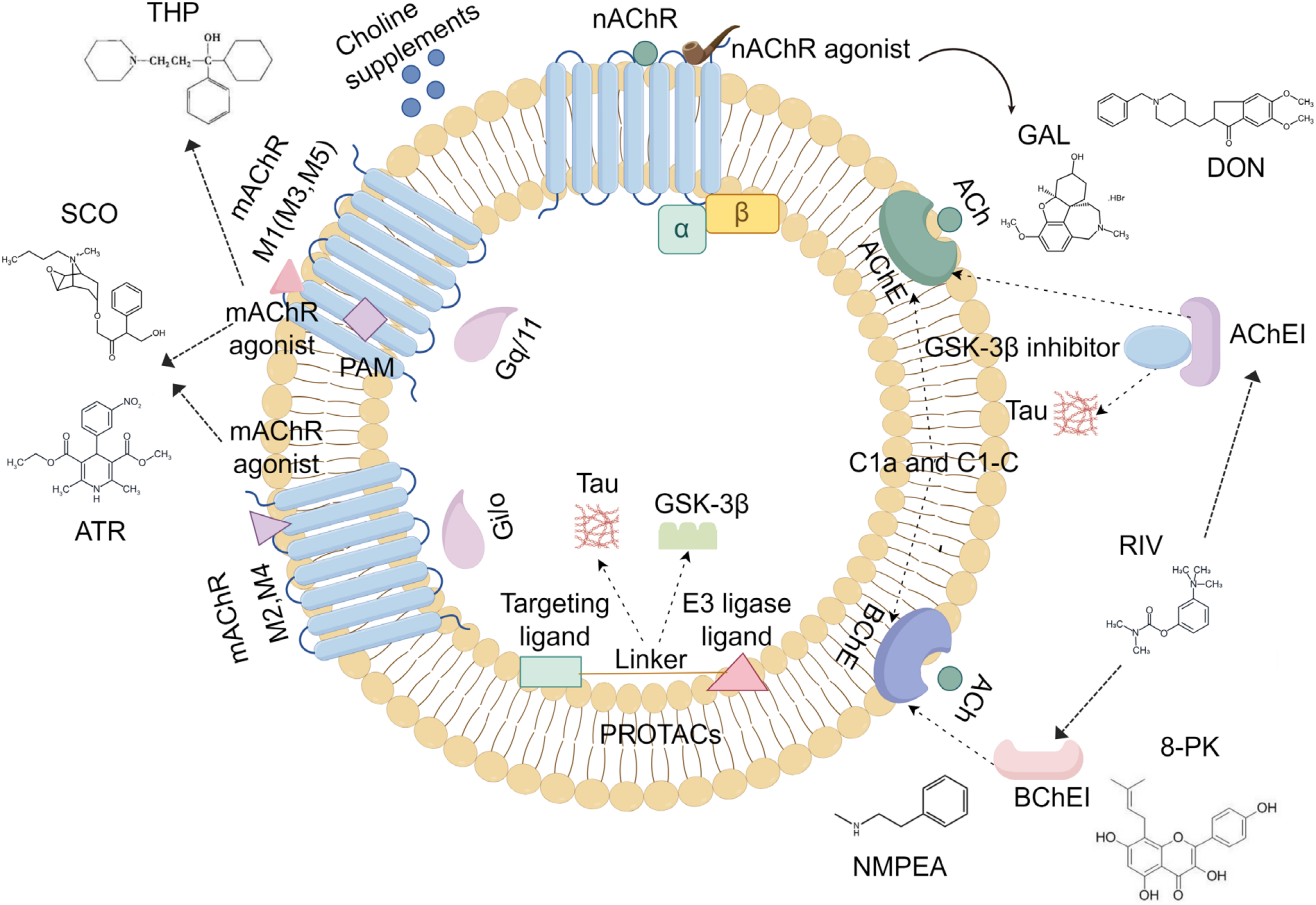
Multi‐target pharmacological interventions in the cholinergic system. This schematic illustrates key therapeutic targets, including nicotinic (nAChR) and muscarinic (mAChR) receptors, acetylcholinesterase (AChE), butyrylcholinesterase (BChE), and downstream targets such as GSK‐3β in tau protein regulation. Dashed arrows indicate inhibitory actions. 8‐PK, 8‐prenylkaempferol; ACh, acetylcholine; AChEI, acetylcholinesterase inhibitor; ATR, atropine; BChEI, butyrylcholinesterase inhibitor; DON, donepezil; GAL, galantamine; NMPEA, N‐methylphenethylamine; PAM, positive allosteric modulator; PROTACs, proteolysis‐targeting chimeras; RIV, rivastigmine (a dual AChE/BChE inhibitor); SCO, scopolamine; THP, trihexyphenidyl.

The recognized limitations of first‐generation AChEIs have served as a critical impetus for the evolution of cholinergic pharmacotherapy [17]. This evolution is characterized by a strategic pivot from the broad enhancement of synaptic acetylcholine toward a more nuanced and sophisticated paradigm. The contemporary pharmacological landscape is now defined by two complementary frontiers: first, the pursuit of precision targeting through the modulation of specific receptor subtypes (nicotinic acetylcholine receptors, nAChRs, and muscarinic acetylcholine receptors, mAChRs) to improve efficacy and minimize side effects [18, 19]; and second, the development of multi‐target directed ligands (MTDLs) designed to concurrently address the multifaceted pathologies of neurodegenerative diseases [20]. This section will chart this progressive journey, examining how innovations ranging from dual‐enzyme inhibitors and receptor‐specific agents to emerging strategies like protein degradation are collectively expanding the therapeutic arsenal beyond conventional single‐target approaches (Figure 1).

### 2.1 AChEIs: Established Efficacy and Inherent Limitations

AChEIs, as a direct product of the cholinergic hypothesis, remain the only class of drugs approved for symptomatic treatment of mild to moderate AD. This class includes donepezil and galantamine [21, 22, 23, 24]. These drugs work by inhibiting the activity of AChE, delaying the breakdown of ACh in the synaptic cleft, and enhancing cholinergic signaling [25]. Clinical studies have shown that AChEIs can improve cognitive function to some extent, particularly in the early stages of AD, although the improvements are typically modest [26]. Long‐term use of AChEIs has been proven to significantly improve cognitive function and may reduce mortality [27]. In addition to cognitive improvement, AChEIs may also inhibit AChE‐induced aggregation of β‐amyloid (Aβ) and reduce the formation of senile plaques, although the clinical significance of this mechanism is still under investigation [28, 29]. Donepezil, as an AChEI, has also been shown to be particularly effective in treating Parkinson's disease‐related dementia (PDD), making it one of the few cholinergic drugs with proven efficacy in this area [30]. In patients with dementia with DLB, randomized controlled trials and subsequent meta‐analyses have supported the beneficial effects of donepezil on cognition and some behavioral symptoms [30, 31].

The combination of AChEIs with N‐Methyl‐D‐Aspartate (NMDA) receptor antagonist memantine has shown additional clinical benefits in moderate to severe AD patients [32]. A meta‐analysis suggests that the dual‐pathway collaboration between the glutamate and cholinergic systems might yield more significant therapeutic effects compared to a single pathway approach [33]. Furthermore, dual inhibitors targeting both AChE and glycogen synthase kinase‐3β (GSK‐3β) are under development, aiming to provide more comprehensive and profound therapeutic effects by simultaneously targeting AChE and GSK‐3β [34]. Natural compounds, such as quercetin, resveratrol, and curcumin, have also gained attention for their potential as AChEI candidates [35, 36, 37]. These polyphenolic compounds have been shown to inhibit AChE activity, with curcumin also possessing antioxidant, anti‐inflammatory, and metabolic regulation properties, making it a promising candidate for AD treatment [38].

However, despite the progress made with AChEIs in improving AD symptoms, these drugs still have significant limitations. Firstly, AChEIs are symptomatic treatments, not disease‐modifying agents, as they do not alter the underlying pathological processes of neurodegenerative diseases such as AD [39]. Secondly, due to neurotransmitter saturation and receptor downregulation, prolonged high levels of ACh stimulation may lead to receptor desensitization, which does not effectively address the insufficient presynaptic ACh synthesis caused by neuronal loss [40]. Additionally, the peripheral cholinergic side effects (such as gastrointestinal reactions) of AChEIs limit dose escalation, thereby reducing treatment effectiveness [41].

Notably, emerging evidence suggests that the benefits of AChEIs may extend beyond symptomatic relief to include neuroprotective effects. Moss and Perez [42] have proposed that AChEIs exert anti‐neurodegenerative benefits by enhancing acetylcholine‐dependent release and uptake of nerve growth factor (NGF). Specifically, they hypothesize that the release of pro‐nerve growth factor (pro‐NGF) is activity‐dependent and regulated by basal forebrain projections to the hippocampus and cortex. By increasing acetylcholine levels, AChEIs augment pro‐NGF release, thereby increasing the availability of mature NGF and supporting the survival of cholinergic basal forebrain neurons, which in turn slows AD‐associated degeneration of the cholinergic basal forebrain and ultimately delays dementia progression [43]. This framework provides a mechanistic link between cholinergic enhancement and the preservation of neural circuits connecting the nucleus basalis of Meynert (NBM) with the entorhinal cortex, hippocampus, and frontal cortex.

Consequently, recent research has shifted toward receptor subtype‐selective modulation and multi‐target strategies. This shift aims to precisely regulate the activation of cholinergic receptor subtypes, avoid the side effects of traditional treatments, and enhance therapeutic efficacy through multi‐target synergism.

### 2.2 Butyrylcholinesterase (BChE) and Dual‐Enzyme Inhibition

As neurodegenerative diseases progress, particularly in late‐stage Alzheimer's disease, the relative contribution of BChE in the cortex increases, providing a new rationale for therapeutic targeting [44]. Since BChE participates in acetylcholine metabolism alongside AChE, interventions targeting BChE have emerged as an important strategy for improving cholinergic neurotransmission dynamics [45]. Recent systematic reviews and experimental studies indicate that BChE inhibitors and dual AChE/BChE inhibitors can effectively restore cholinergic balance in both in vitro and in vivo models, offering potential treatment options particularly for advanced‐stage patients who have become tolerant to AChEIs [46, 47, 48].

Notably, rivastigmine, an established dual AChE/BChE inhibitor, is already in routine clinical use for the treatment of mild to moderate Alzheimer's disease and Parkinson's disease dementia [49]. Unlike donepezil, which selectively inhibits AChE, rivastigmine acts as a pseudo‐irreversible inhibitor of both AChE and BChE, offering a broader spectrum of cholinesterase inhibition [50]. Its dual inhibitory profile may be particularly advantageous in advanced disease stages, where BChE activity becomes relatively more prominent in the cortex and may compensate for declining AChE function [51]. Clinical evidence supports the efficacy of rivastigmine in improving cognitive function and global clinical status, and its transdermal patch formulation provides a favorable tolerability profile with reduced gastrointestinal side effects compared to oral administration [49, 52]. The clinical success of rivastigmine validates the dual‐enzyme inhibition strategy and has encouraged further exploration of novel dual inhibitors with improved selectivity and safety profiles.

The integration of computational modeling and virtual screening has accelerated the discovery of natural product‐derived inhibitors. Researchers have identified several potent BChE inhibitors from natural plant compounds, including 8‐prenylkaempferol and Na‐methylisobutylamine [53]. These compounds not only demonstrate strong BChE inhibitory activity but also possess multiple biological properties such as antioxidant and anti‐inflammatory effects, enhancing their potential therapeutic value for neurodegenerative diseases [54].

Significant progress has also been made in developing novel dual AChE/BChE inhibition strategies beyond rivastigmine. Recent studies show that certain organoammonium (II) complexes, such as C1a and C1‐C, can simultaneously inhibit both AChE and BChE with significant dual inhibitory activity [38]. These compounds improve cholinergic function through dual mechanisms, potentially overcoming the limitations of traditional AChEIs. To accelerate the discovery of novel inhibitors, researchers have developed machine learning models that can rapidly screen promising compounds by analyzing chemical structures and biological activity data, further advancing the development of dual‐enzyme inhibitors and multi‐target molecules [55]. This multi‐target approach not only enhances cholinergic signaling but also provides more therapeutic possibilities for late‐stage neurodegenerative diseases. It should be noted, however, that the independent clinical benefit of selective BChE inhibitors (as opposed to dual AChE/BChE inhibitors) in AD patients has not been confirmed by any Phase III trial; their therapeutic standing remains largely based on the pathological assumption of compensatory upregulation of BChE activity.

### 2.3 nAChRs: Subtype‐Selective Targeting Challenges

To overcome the lack of receptor subtype selectivity and potential receptor desensitization associated with AChEIs, research has shifted toward agonists targeting specific nAChR subtypes [56]. nAChRs are ligand‐gated ion channel receptors involved not only in neurotransmitter release and synaptic plasticity but also in neuroinflammation and microglial responses [57]. Different nAChR subtypes serve distinct functions in the nervous system, with the α7 nAChR receiving particular attention as a dual‐purpose cognitive‐anti‐inflammatory target due to its role in regulating neuroinflammation and protecting neurons [58, 59]. Additionally, the α4β2 subtype is closely associated with attention and working memory, making it an important target for cognitive impairment treatment [60].

EVP‐6124, a selective α7 nAChR agonist, initially showed potential for improving cognitive function in Alzheimer's disease patients in early clinical trials. However, the drug was discontinued in Phase III trials due to severe gastrointestinal adverse effects [61]. Although encenicline demonstrated neuroprotective, anti‐inflammatory, and cognitive‐enhancing potential in preclinical studies, its repeated failures in clinical applications reveal a fundamental problem: as the disease progresses, the loss of cholinergic neurons—particularly the significant reduction of nAChRs on their axonal terminals—renders agonist therapies ineffective, creating a missing target scenario [62]. This represents a major challenge in their clinical application.

Notably, galantamine exhibits somewhat different clinical characteristics compared to other AChEIs. This may be partially attributed to its unique dual mechanism. Unlike traditional AChEIs such as donepezil, galantamine not only inhibits AChE but also acts as a positive allosteric modulator of nAChRs [63]. This characteristic enables it to enhance endogenous ACh effects rather than directly activating receptors, potentially providing more physiological, spatiotemporally specific signal enhancement within residual cholinergic circuits [64]. This may explain its distinct clinical profile and offers important insights for future drug design.

### 2.4 mAChRs and M1 Positive Allosteric Modulators (PAMs)

mAChRs belong to the G protein‐coupled receptor (GPCR) family and are divided into five subtypes (M1‐M5). The M1, M3, and M5 subtypes couple with Gq/11 proteins and mediate excitatory signaling, while the M2 and M4 subtypes couple with Gi/o proteins and primarily mediate inhibitory signaling [25]. mAChRs are widely distributed throughout the central nervous system, with the M1 subtype highly expressed in postsynaptic regions of the cerebral cortex and hippocampus, making it a key target for restoring cognitive network excitation‐inhibition balance [65]. Consequently, the M1 subtype has become a primary target for restoring cognitive function in neurodegenerative diseases. The M2 subtype is mainly located presynaptically and regulates acetylcholine release, while the M4 subtype is abundantly expressed in the striatum and influences motor control and other nervous system functions by modulating dopamine release [66, 67].

It is important to note that long‐term use of antimuscarinic drugs (such as atropine, scopolamine, and trihexyphenidyl) has been proven to be closely associated with increased dementia risk [68, 69, 70], highlighting the potential harm of anticholinergic burden to the aging brain and further emphasizing the need for selective M1/M4 modulation.

Traditional mAChR agonists are often limited by significant side effects due to their lack of subtype selectivity, representing a major bottleneck in current treatment approaches. This challenge has prompted the development of PAMs as a new drug strategy. M1 mAChR PAMs can highly selectively target the M1 subtype by binding to allosteric sites on M1 mAChRs, inducing conformational changes that increase the affinity or efficacy of acetylcholine without directly activating the receptor [71].

Preclinical studies demonstrate that M1 mAChR PAMs (such as VU0486846 and VU0453595) significantly improve cognitive function, reduce Aβ oligomer levels, and normalize sleep–wake architecture in Alzheimer's disease models like APP/PS1 mice, while avoiding cholinergic‐like toxicity side effects. These findings highlight the potential of M1 mAChR PAMs in AD treatment, representing an important advancement in pharmacology [72, 73, 74, 75]. Additionally, TAK‐071, a novel M1 PAM, reduces falls in cholinergic‐dopaminergic deficiency models and improves attention in pure cholinergic deficit models [76]. These preclinical successes have prompted a series of M1 PAM candidates to enter clinical development. However, clinical translation has proven challenging. For instance, MK‐7622 (Merck), one of the most advanced M1 PAMs, failed to improve cognition in a Phase II trial in mild‐to‐moderate AD patients, with 16% of treated patients experiencing cholinergic adverse effects such as diarrhea—attributed to its robust intrinsic agonist activity and on‐target M1 activation [77, 78]. Similarly, PF‐06767832 (Pfizer), a highly selective M1 PAM‐agonist, reversed cognitive deficits in rodent models but induced convulsions as well as gastrointestinal and cardiovascular adverse effects in rats, effects shown to be on‐target rather than off‐target [79]. Although most candidates have failed due to narrow therapeutic windows or unexpected side effects, these efforts nevertheless validate the therapeutic potential of this target and guide the design of next‐generation, safer compounds—particularly those with low intrinsic agonist activity (“pure PAMs”) to minimize on‐target cholinergic toxicity.

### 2.5 Multi‐Target Directed Ligands

As the limitations of single‐target therapies become increasingly apparent, the treatment of neurodegenerative diseases is moving toward multi‐target strategies. By integrating actions on different targets, drugs can simultaneously address multiple pathological issues, significantly improving therapeutic efficacy. AChE/GSK‐3β dual inhibitors represent this trend. These compounds are designed by combining the core structures of AChE inhibitors with GSK‐3β inhibitors, simultaneously alleviating cholinergic deficits and reducing tau protein hyperphosphorylation, thereby effectively clearing neurofibrillary tangles and improving cognitive function [80]. This multi‐target approach addresses the limitations of single‐target therapies and provides more comprehensive treatment effects.

Similarly, ladostigil, a dual AChE and monoamine oxidase‐B (MAO‐B) inhibitor, has demonstrated good tolerability and potential for delaying Alzheimer's disease progression in Phase II clinical trials. By simultaneously inhibiting AChE and MAO‐B, ladostigil not only increases acetylcholine levels but also reduces neurotransmitter breakdown, comprehensively improving neurotransmission [81]. This multi‐target strategy holds the potential to provide broader efficacy through simultaneous modulation of multiple pathological pathways [82]. However, it is important to note that the evidence supporting superior clinical efficacy over single‐target agents or reduced risk of drug resistance remains largely preclinical. To date, no multi‐target directed ligand (MTDL) has demonstrated superior efficacy over established single‐target drugs, such as donepezil, in a rigorous, large‐scale randomized controlled trial. Indeed, most multi‐target compounds have not yet been rigorously compared with established single‐target drugs in head‐to‐head clinical trials, and their theoretical advantages require validation through well‐designed, adequately powered Phase III studies before any definitive conclusions can be drawn regarding their clinical superiority [83, 84].

### 2.6 Cholinergic Supplementation

As an important neurotransmitter precursor, choline supplementation has gained increasing attention in recent years for its potential application in mild cognitive impairment (MCI) and some Alzheimer's disease patients [70, 85, 86]. Choline precursors such as α‐GPC have shown some improvement signals in these patient populations, particularly in cognitive function. However, despite preliminary positive results, current evidence remains inconsistent, necessitating more rigorous randomized controlled trials (RCTs) to confirm their efficacy and clinical application value [87].

Meanwhile, epidemiological studies suggest that higher dietary choline intake is associated with reduced dementia risk, though establishing clear causality remains challenging due to confounding factors [88]. Recent gut‐brain axis research has provided new perspectives on the relationship between cholinergic systems and cognitive function. New evidence indicates that certain gut bacteria, such as *Bacteroides ovatus* , and their metabolites including lysophosphatidylcholine (LPC), can significantly reduce Aβ burden and improve cognitive function in 5xFAD mouse models [89, 90]. These findings suggest complex interactions between gut microbiota and choline metabolism, indicating that gut microorganisms may become new intervention targets for neurodegenerative diseases through modulation of the lipid‐choline metabolism–neuroinflammation pathway [91, 92, 93, 94].

Overall, drug therapy is gradually evolving from traditional ACh enhancement strategies toward precise receptor/pathway regulation and multi‐target synergy. However, although these drugs can improve symptoms to some extent, they still cannot fully reconstruct degenerated cholinergic projections or repair damaged neural circuits, particularly in late disease stages. Therefore, the limitations of pharmacological approaches are driving the field toward a new phase of neuromodulation‐drug integration.

## 3 Neuromodulation: From Circuit Engagement to Network Repair

**FIGURE 2:**
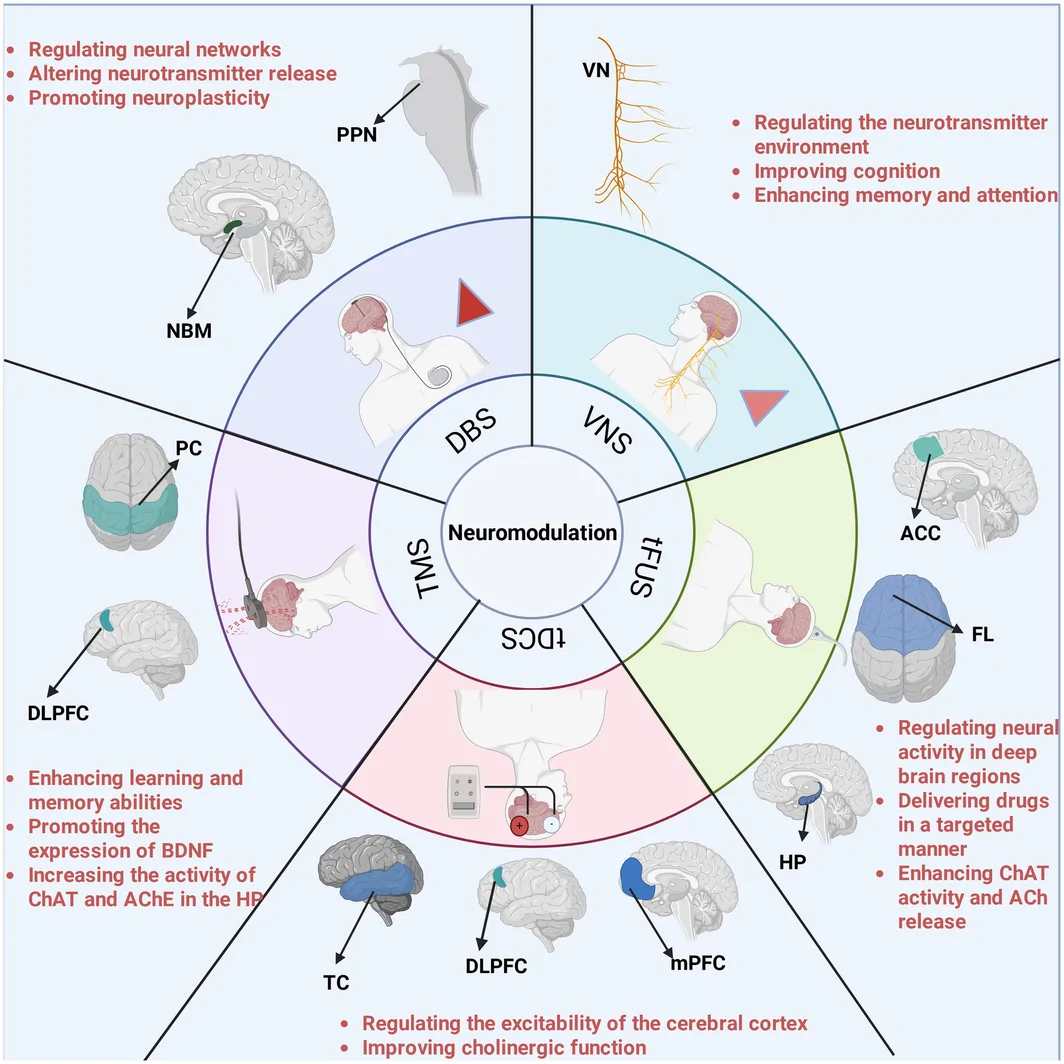
Comparison of five neuromodulation techniques. Key differentiating features include the mechanism of stimulation, primary target sites in the nervous system, associated functions, and degree of invasiveness (visualized via triangles, with color intensity proportional to invasiveness). Abbreviations for relevant neural structures are listed. ACC, anterior cingulate cortex; DBS, deep brain stimulation; DLPFC, dorsolateral prefrontal cortex; FL, frontal lobe; HP, hippocampus; mPFC, medial prefrontal cortex; NBM, nucleus basalis of Meynert; PC, parietal Cortex; PPN, pedunculopontine nucleus; TC, temporal cortex; tDCS, transcranial direct current stimulation; tFUS, transcranial focused ultrasound stimulation; TMS, transcranial magnetic stimulation; VN, vagus nerve; VNS, vagus nerve stimulation.

As traditional pharmacological therapies face limitations, the treatment of neurodegenerative diseases has begun exploring direct neural circuit interventions to repair function [95]. The core advantage of neuromodulation technologies lies in their ability to bypass neurotransmitter systems and directly target neuronal electrical activity and network rhythms, potentially reshaping the functional state of entire neural circuits [19]. This strategy provides new approaches for treating neurodegenerative diseases, demonstrating significant potential particularly where drug efficacy is limited (Figure 2).

### 3.1 Vagus Nerve Stimulation/Transcutaneous Vagus Nerve Stimulation (VNS/tVNS)

The vagus nerve, the longest nerve in the autonomic nervous system, extensively regulates physiological processes such as heart rate, blood pressure, and digestion, while also influencing cognitive processing in the brain [96]. tVNS modulates the brainstem nucleus tractus solitarius by stimulating the peripheral vagus nerve, subsequently affecting the locus coeruleus (noradrenergic) and raphe nuclei (serotonergic), and ultimately regulating the whole‐brain neurotransmitter environment and synaptic plasticity through synergistic effects [97]. This mechanism of action is network‐based rather than single‐target stimulation.

In clinical research, tVNS has gained support from systematic reviews and multiple trials for improving cognitive functions such as memory and attention in healthy populations and elderly subjects [98, 99, 100]. Specifically, tVNS effectively enhances associative memory in healthy older adults, strengthens emotional memory consolidation, improves spatial working memory, and demonstrates significant effects in emotion regulation and attention [101, 102, 103, 104, 105, 106, 107]. For example, tVNS application in emotional conflict processing enhances individual cognitive control ability and improves the capacity to filter relevant information in noisy environments during auditory selective attention tasks [100, 108, 109, 110]. Systematic reviews and meta‐analyses have also confirmed the positive effects of tVNS in healthy individuals, particularly regarding memory and attention enhancement [111]. However, it must be noted that most of these benefits have been demonstrated in healthy volunteers or patients with mild cognitive impairment (MCI); evidence in patients with a clear diagnosis of Alzheimer's disease or Parkinson's disease dementia remains limited and inconsistent [112, 113, 114]. Notably, tVNS exhibits low‐risk, stackable characteristics, making it particularly suitable for developing personalized treatment protocols, with potential for combined application with cognitive training and pharmacological treatments to further enhance efficacy. Future research will likely focus on optimizing individualized treatment protocols and exploring its combined application with other therapeutic approaches.

### 3.2 Deep Brain Stimulation (DBS)

In the mammalian central nervous system, cholinergic neurons can be categorized into three main types: motor neurons, interneurons, and projection neurons, with cholinergic projection neurons primarily including basal forebrain cholinergic neurons (NBM) and pedunculopontine nucleus (PPN) cholinergic neurons [115, 116, 117]. DBS, a therapeutic method involving implanted electrodes to stimulate specific brain regions, has been widely used in treating neurodegenerative diseases, showing significant potential particularly in circuit node repair and neural network remodeling. DBS functions by regulating neural networks, altering neurotransmitter release, and promoting neuroplasticity, with DBS targeting the cholinergic system drawing particular research interest [118].

Different cholinergic nuclei are responsible for distinct functions, necessitating precise targeted therapy. In AD, NBM‐DBS aims to awaken residual basal forebrain cholinergic neurons through electrical stimulation, enhancing their cortical projections to increase global cholinergic tone in a top‐down manner. This treatment extends beyond merely increasing neurotransmitter release to promoting the secretion of neurotrophic factors and enhancing neural network plasticity [119]. Early studies and recent Phase I clinical trials have demonstrated the potential of NBM low‐frequency stimulation for safety and cognitive improvement, with efficacy comparable to conventional treatments. Imaging and anatomical subtype analyses indicate that treatment benefits may depend on the structural integrity and connectivity preservation of the frontoparietal network, emphasizing the necessity for population stratification and individualized targeting.

In PD, the PPN primarily controls gait and posture [120, 121]. PPN‐DBS aims to modulate the brainstem‐spinal cord motor pathway in a bottom‐up manner, which is crucial for gait and postural control [120, 122]. Although clinical studies show heterogeneous effectiveness of PPN‐DBS across different patients, consistent conclusions emphasize the critical importance of electrode placement and frequency parameters in treatment efficacy. Recent consensus and reviews highlight the PPN's hub role in regulating sleep–wake cycles, posture–gait, and cortical gamma rhythms, establishing it as a key network target for late‐stage PD gait disorders [123, 124]. With a deepening understanding of cholinergic system functions and continuous optimization of stimulation parameters, the application prospects of DBS in treating neurodegenerative diseases are expanding. A summary of key clinical trials and their outcomes is presented in Table 1.

**TABLE 1:** Clinical trials of the treatment of neurodegenerative diseases with DBS.

References	Target	Disease	Intervention		*N*	Results
			T	C		
Jiang et al. [125]	NBM	AD	Active stimulation	Sham stimulation	8	NBM‐DBS improves short‐term cognitive performance in patients with advanced AD
Xu et al. [126]	NBM	AD	Active stimulation	N/A	6	It improved cognitive functions and self‐care abilities
Xu et al. [127]	Fx&NBM	AD	Active stimulation	N/A	20	NBM‐DBS showed distinct advantages in ameliorating neuropsychiatric symptoms
Kuhn et al. [128]	NBM	AD	Active stimulation	N/A	6	DBS of the NBM is both technically feasible and well tolerated
Cappon et al. [129]	NBM	PDD	Active stimulation	N/A	5	The conditions of the two patients progressed relatively slowly during the three‐year follow‐up period
Dayal et al. [130]	PPN	PD&PSP	Bilateral electrode implantation	Single‐side electrode implantation	6	Bilaterally treated patients showed improvements in the gait questionnaire, UPDRS‐III, PSPRS, and their gait sub‐items
Wilcox et al. [131]	PPN	PD	Active stimulation	N/A	1	Patients showed significant improvements in gait and posture after intervention
Plaha et al. [132]	PPN	PD	Active stimulation	N/A	2	This stimulation significantly improves patients' gait disturbances and postural instability
Servello et al. [133]	PPN&GPi	PSP	DBS of PPN and GPi	DBS of PPN	3	Combined target stimulation better improves gait and reduces fall events over time compared with DBS of a single target
Thevathasan et al. [134]	PPN	PD	Unilateral stimulation or bilateral stimulation	Sham stimulation	7	PPN stimulation improved objective gait freezing measures, with bilateral stimulation more effective than unilateral
Mestre et al. [135]	PPN	PD	Active stimulation	Sham stimulation	9	Pedunculopontine area stimulation has an initial but not sustained benefit for gait‐related symptoms
Moro et al. [136]	PPN	PD	Active stimulation	Sham stimulation	6	PPN‐DBS may be effective in preventing falls in patients with advanced PD
Nosko et al. [137]	PPN	PD	Low‐frequency stimulation	Higher frequency stimulation	9	Low‐frequency stimulation better improves patients' gait and reduces postural disorders than high‐frequency stimulation

Abbreviations: AD, Alzheimer's disease; C, control group (sham/placebo); DBS, deep brain stimulation; Fx, Fornix; GPi, globus pallidus pars interna; *N*, number of participants; NBM, nucleus basalis of Meynert; PD, Parkinson's disease; PDD, Parkinson's disease dementia; PPN, pedunculopontine nucleus; PSP, progressive supranuclear palsy; PSPRS, progressive supranuclear palsy rating scale; T, treatment group; UPDRS‐III, Unified Parkinson's Disease Rating Scale Part III.

### 3.3 Non‐Invasive Brain Stimulation (NIBS)

Non‐invasive neuromodulation techniques, including transcranial magnetic stimulation (TMS), transcranial direct current stimulation (tDCS), and transcranial focused ultrasound stimulation (tFUS), provide novel therapeutic approaches for neurodegenerative diseases [119, 138]. These techniques modulate neural network functional states through different mechanisms of action without the need for surgery or pharmacological agents.

TMS and tDCS indirectly influence the cortex‐basal forebrain circuit through cortical entry points, thereby regulating whole‐brain neural activity. Evidence indicates that these techniques effectively modulate cortical excitability and may enhance basal forebrain cholinergic neuron (BFCN) function through retrograde signaling [139, 140]. In animal models of vascular dementia and AD, repetitive TMS has been shown to increase hippocampal ChAT and AChE activity, improve learning and memory capacity, increase cholinergic fiber density, and promote brain‐derived neurotrophic factor expression [139]. However, the effects of TMS on ChAT are region‐ and protocol‐dependent, indicating the need for further optimization of stimulation parameters to maximize clinical efficacy. In human studies, multiple randomized controlled trials and systematic reviews have demonstrated that repetitive TMS (rTMS) can improve global cognitive function in patients with mild MCI and AD [141]. Pharmaco‐TMS‐EEG studies—a research paradigm that combines pharmacological interventions with concurrent TMS and electroencephalography (EEG) to directly measure drug effects on cortical excitability, connectivity, and network dynamics—further reveal that TMS effects are modulated by the cholinergic system. For instance, recent placebo‐controlled pharmaco‐TMS‐EEG studies using muscarinic acetylcholine receptor (mAChR) antagonists have demonstrated that cholinergic neurotransmission contributes to TMS‐evoked EEG potentials in a target‐specific manner, providing strong evidence for using TMS as a non‐invasive readout tool for cholinergic function [142].

Nevertheless, the clinical translation of TMS and tDCS faces substantial challenges. For TMS, marked inter‐individual variability in responses, the lack of standardized optimal stimulation frequency, intensity, target site, and treatment regimen, and the relatively short duration of after‐effects—requiring repeated sessions to sustain benefit—remain unresolved. Moreover, the effect sizes on cognitive outcomes are generally modest, and the direct evidence for cholinergic mechanisms in humans is still incomplete. For tDCS, the electric field distribution in the brain is diffuse, limiting precise targeting of specific regions or nuclei; effect sizes are small and highly heterogeneous, with inconsistent results across trials; and optimal stimulation parameters (electrode montage, current intensity, duration) have not been standardized, hindering comparability across multicentre studies. In addition, the indirect impact of tDCS on deep cholinergic nuclei via cortical modulation is not fully elucidated in humans [143, 144].

Compared with TMS/tDCS, tFUS offers unique advantages, including high spatial precision (millimeter‐scale) and the ability to reach deep brain structures [145, 146]. tFUS can simultaneously modulate neuronal activity and non‐invasively open the blood–brain barrier, enabling targeted drug delivery [147, 148]. Using MRI‐guided FUS, agonists of tropomyosin receptor kinase A (TrkA) can be accurately delivered to the basal forebrain, significantly increasing ChAT activity and ACh release in AD models and improving behavioral performance [149]. Standalone tFUS has also been shown to promote neuroplasticity recovery after injury [150].

However, the translational pathway for tFUS is hindered by several critical barriers. Direct evidence in the human cholinergic system is currently lacking, with most data derived from animal models [151]. The effects of tFUS on neuronal excitability—whether excitatory or inhibitory—depend on a complex interplay of parameters (acoustic intensity, frequency, duty cycle, pulse repetition frequency, etc.) that are not yet fully understood [152, 153]. Long‐term safety data, especially concerning repeated blood–brain barrier opening, are insufficient [151]. The high cost and operational complexity of the equipment limit widespread clinical adoption, and optimal stimulation parameters have not been standardized, leading to considerable variability across studies and complicating the comparison of outcomes and effect sizes [152, 153]. Despite these obstacles, tFUS remains a promising approach, particularly as a potential conduit for combining physical modulation with pharmacological therapy [151, 153].

In conclusion, neuromodulation technologies signify a paradigm shift from traditional chemical enhancement to physical modulation in treating neurodegenerative diseases. Their core objective is no longer simply to increase the concentration of specific neurotransmitters, but to reset aberrant brain network states by modulating neural activity, thereby restoring intrinsic information processing capacity [154]. Unlike pharmacological treatments that primarily rely on neurotransmitter modulation, neuromodulation compensates drug therapy by directly acting on neuronal electrical activity and network rhythms in—effectively aiming at restoring neural projections and remodeling network dynamics [155]. Nevertheless, the clinical realization of advanced concepts such as imaging‐guided closed‐loop systems remains at an early exploratory stage, with numerous technical and validation hurdles to overcome before routine application becomes feasible. With continued refinement and standardization of these techniques, neuromodulation is expected to be integrated with pharmacological treatments, opening new therapeutic horizons for neurodegenerative diseases [148]. Key clinical trial outcomes and related findings are summarized in Table 2.

**TABLE 2:** Clinical trials of the treatment of neurodegenerative diseases with VNS and NIBS.

References	Target	Disease	Intervention		*N*	Results
			T	C		
Cheng et al. [156]	DLPFC	PD	rTMS‐VR or rTMS	Sham rTMS	40	The integrated rTMS‐VR protocol achieved a superior outcome in global cognitive function
Marron et al. [157]	DLPFC&PC	PD	Active TMS	Sham TMS	54	It is expected to improve memory and enhance cognitive abilities
Boggio et al. [158]	DLPFC& TC	AD	Active tDCS	Sham tDCS	10	tDCS stimulation can enhance a component of recognition memory
Andrade et al. [159]	DLPFC	AD	Active tDCS + CS	Sham tDCS + CS	36	Anodal tDCS + CS improved overall cognitive function and changed EEG brain activity
Gonzalez et al. [160]	DLPFC	MCI	tDCS + CT	Sham tDCS + CT	67	The tDCS + CT group showed greater improvement in overall cognitive ability and daily memory capacity
LoBue et al. [161]	mPFC	AD	tDCS	N/A	25	Some Alzheimer's disease patients show significant improvement in cognitive ability
Qin et al. [162]	HP&FL	PD	tFUS	N/A	20	The load of β‐amyloid protein plaques decreased
Jeong et al. [163]	HP	AD	tFUS	N/A	4	The patient's cerebral glucose metabolism and cognitive function are improved
Nicodemus et al. [164]	HP or SN	AD&PD	tFUS	N/A	22	62.5% of patients had one or more improved cognitive scores
Ye et al. [165]	BFL	AD	tFUS	N/A	6	Improving neurological and mental symptoms by opening the blood–brain barrier
Meng et al. [166]	HP&ACC	AD	tFUS	N/A	9	It failed to significantly improve cognitive abilities, but is expected to be used in combination with other medications for treatment
Murphy et al. [99]	VN	MCI	tVNS	N/A	50	tVNS alters the activity patterns of brain networks associated with lesion progression
Wang et al. [100]	VN	MCI	taVNS	sham taVNS	52	taVNS can improve cognitive performance in patients with MCI
Merrill et al. [167]	VN	AD	VNS	N/A	17	VNS therapy exerts a positive impact on the cognitive function of patients with AD

Abbreviations: ACC, anterior cingulate cortex; AD, Alzheimer's disease; BFL, bilateral frontal lobes; C, control group (sham/placebo); DLPFC, dorsolateral prefrontal cortex; EEG, electroencephalography; FL, frontal lobe; HP, hippocampus; MCI, mild cognitive impairment; mPFC, medial prefrontal cortex; *N*, number of participants; PC, parietal cortex; PD, Parkinson's disease; rTMS, repetitive transcranial magnetic stimulation; SN, substantia nigra; T, treatment group; taVNS, transcutaneous auricular vagus nerve stimulation; TC, temporal cortex; tDCS, transcranial direct current stimulation; tFUS, transcranial focused ultrasound stimulation; TMS, transcranial magnetic stimulation; tVNS, transcutaneous vagus nerve stimulation; VN, vagus nerve; VNS, vagus nerve stimulation; VR, virtual reality.

## 4 Imaging and Biomarkers: Visualizing Cholinergic Integrity

The rapid development of biomarkers and imaging technologies in recent years has provided powerful tools for in vivo assessment of the cholinergic system, playing a crucial role in the diagnosis, disease monitoring, and treatment evaluation of neurodegenerative diseases [168]. Positron emission tomography (PET) and single‐photon emission computed tomography (SPECT) are currently the primary techniques for visualizing cholinergic targets in the brain, offering high sensitivity and non‐invasive characteristics that provide precise data support for functional assessment of the cholinergic system [169, 170, 171]. Second‐generation PET tracers, particularly the vesicular acetylcholine transporter (VAChT) tracer [^18^F]‐FEOBV, can directly reflect cholinergic terminal density and quantitatively evaluate the distribution of cholinergic nerve terminals [172, 173]. Additionally, tracers such as [^11^C]MP4A and [^11^C]PMP can assess AChE activity, while nAChR and mAChR subtype‐specific ligands form a complementary imaging system, collectively providing multidimensional data for quantitative analysis of cholinergic receptors [174, 175]. These techniques offer profound spatial topological information about cortical denervation in diseases like AD and dementia with DLB, and directly guide treatment decisions. For example, patients showing severe cholinergic deficits on VAChT‐PET may respond poorly to AChEIs but could be potential candidates for NBM‐DBS or cholinergic cell transplantation [176, 177].

Despite these advances, the widespread clinical implementation of cholinergic PET imaging faces several substantial hurdles. The production and availability of specialized PET tracers, particularly [^18^F]‐FEOBV, remain limited to a few research centres due to complex synthesis requirements and high costs [178], which severely restricts their use in routine clinical practice and multicentre trials. Standardization of acquisition protocols, image reconstruction, and quantitative analysis across different PET scanners and sites has not been achieved, complicating cross‐study comparisons and the establishment of reliable normative databases [178, 179]. Moreover, PET imaging necessitates exposure to ionizing radiation, which limits its use for repeated longitudinal assessments in the same patient and precludes application in certain populations. Additionally, current tracers provide static measurements of cholinergic marker density or enzyme activity but cannot capture the dynamic fluctuations in acetylcholine release or receptor occupancy that occur in real time [178]. The interpretation of PET data is further confounded by age‐related physiological changes in cholinergic markers and by the coexistence of multiple pathologies in elderly populations [179, 180], making it difficult to attribute observed changes specifically to a single disease process.

Structural MRI and diffusion tensor imaging (DTI) are also used to track atrophy of the basal forebrain (Ch4/NBM) and white matter integrity, which not only helps predict clinical conversion trajectories in AD patients but also provides strong prognostic markers for disease progression [181]. The integration of imaging technologies and biomarkers promotes the realization of individualized interventions, serving not only for pathological stratification but also providing the basis for companion assessments in pharmacological treatments and neuromodulation [182]. However, the clinical utility of structural and diffusion imaging is constrained by several factors. Segmentation of the basal forebrain cholinergic nuclei on conventional MRI remains technically challenging due to their small size and ill‐defined boundaries, with significant variability across different automated segmentation algorithms. DTI metrics, while sensitive to white matter microstructural changes, lack specificity for cholinergic pathways and are influenced by a wide range of non‐cholinergic pathological processes, including vascular damage, inflammation, and glial changes [10]. Furthermore, standardized imaging protocols and quality control measures are not uniformly applied across centres, and the prognostic value of these imaging biomarkers in individual patients is not yet sufficiently robust to guide clinical decision‐making independently [10, 183].

Furthermore, small non‐coding RNAs such as microRNAs have gained widespread attention in recent years as peripheral biomarkers of cholinergic dysfunction [184, 185, 186]. These biomarkers show great potential in predicting disease progression, treatment response, and evaluating drug efficacy. By combining clinical markers (such as cognitive tests, gait analysis) with imaging techniques, patient populations with cholinergic deficits can be more accurately identified, thereby providing stronger evidence for early intervention and personalized treatment. Nevertheless, the translation of microRNA biomarkers to clinical practice is hampered by several important limitations. The tissue specificity of circulating microRNAs is poor [187], and their expression levels can be affected by numerous confounding factors including age, sex, comorbidities, medications, and dietary status [188]. Inter‐study variability in microRNA detection and quantification methods is high, and there is currently no consensus on reference genes or normalization strategies, leading to inconsistent results across different cohorts [187, 188]. Importantly, the causal relationship between altered circulating microRNA levels and central cholinergic dysfunction remains unclear, and whether these changes reflect a primary pathogenic process or a secondary epiphenomenon is unknown. Large‐scale, prospective, longitudinal studies are still needed to validate their predictive value and clinical utility before they can be integrated into routine diagnostic or monitoring algorithms [187, 189].

In summary, the combination of imaging technologies and biomarkers is driving continuous advancement in the diagnosis, monitoring, and treatment evaluation of cholinergic‐related diseases. However, the integration of multimodal imaging and targeted technologies into routine clinical practice must overcome substantial technical, logistical, and validation challenges. Rather than heralding an imminent era of personalized closed‐loop therapy, the current evidence suggests that these tools are most valuable for research purposes and for stratifying patients in clinical trials [190]. Achieving robust, standardized, and widely accessible biomarker‐guided approaches will require coordinated multicentre efforts, harmonized protocols, and large prospective validation studies, which are likely to take many years to materialize. Nevertheless, continued progress in this field holds the potential to refine patient selection, improve trial efficiency, and ultimately inform more individualized clinical decision‐making in neurodegenerative diseases.

## 5 Gene and Cell Strategies: From Trophic Support to Replacement

Gene therapy and cell replacement therapies provide novel strategies for fundamentally restoring or protecting cholinergic function in neurodegenerative diseases. Gene therapy uses viral vectors (such as adeno‐associated virus, AAV) to deliver specific genes into cholinergic neurons or target areas, aiming to support the survival of residual cholinergic neurons and promote functional recovery [191]. For example, delivering genes for neurotrophic factors (such as nerve growth factor, NGF, and brain‐derived neurotrophic factor, BDNF) to the basal forebrain via AAV vectors can enhance the survival capacity of cholinergic neurons and promote axonal regeneration, thereby providing survival support for the treatment of degenerative diseases [192, 193]. This strategy represents a shift from simple neurotransmitter enhancement toward providing trophic support, seeking to slow the progression of neurodegenerative diseases through genetic repair mechanisms. Early AAV2‐NGF gene therapy clinical trials have demonstrated long‐term expression safety, but cognitive improvement effects have been modest at best. This suggests that although gene therapy offers promise for treating neurodegenerative diseases, delivery methods and therapeutic efficacy still require substantial optimization. For instance, using MRI‐guided convection‐enhanced delivery (CED) can improve the targeting and delivery efficiency of gene vectors, potentially enhancing treatment outcomes [194, 195].

Despite its theoretical promise, the clinical translation of gene therapy for cholinergic dysfunction faces numerous formidable barriers. Viral vector‐mediated gene delivery, particularly using AAV, is limited by packaging capacity constraints (approximately 4.7 kb for AAV), which restricts the size of genetic constructs that can be delivered. The immunogenicity of viral vectors remains a major concern, as pre‐existing neutralizing antibodies in the human population can significantly reduce transduction efficiency, while vector‐induced immune responses may cause inflammation and limit long‐term transgene expression [196]. Furthermore, achieving sufficient distribution of vector throughout targeted brain regions is challenging; convection‐enhanced delivery, while improving local distribution, remains an invasive neurosurgical procedure with inherent risks and is not readily scalable [197]. The specificity of gene expression is another critical issue: standard promoters may drive expression in non‐target cell types, potentially leading to off‐target effects, and achieving cell‐type‐specific expression in cholinergic neurons requires carefully engineered promoters that have not been fully validated in humans. Long‐term safety data on AAV‐mediated gene expression are still limited, and there are theoretical concerns about insertional mutagenesis, although these are less pronounced with AAV than with integrating vectors [198, 199]. The high cost of vector production and quality control, coupled with the complex regulatory pathway for gene therapy products, presents substantial economic and logistical barriers to widespread clinical adoption.

Developing in parallel with gene therapy, cell replacement therapy involves differentiating embryonic stem cells or induced pluripotent stem cells (iPSCs) into cholinergic neurons and transplanting them into lesioned brain areas, achieving true structural repair [200, 201]. Studies have shown that transplantation of neural stem cells or mesenchymal stem cells can restore cholinergic neuron numbers, increase ChAT expression, and improve cognitive function in animal models [202]. Differentiation protocols using growth factors, small molecule compounds, or genetic modifications can effectively promote the directed differentiation of stem cells into cholinergic‐like neurons [203, 204, 205]. Strategies combining gene therapy with cell transplantation demonstrate synergistic effects. For example, using engineered stem cells that express neurotrophic factors significantly improves neuronal survival, integration, and functional recovery [206]. Additionally, cholinergic precursor cells derived from human chorion or iPSCs exhibit strong integration capacity and can effectively improve cognitive function, further advancing the potential of neural repair [203, 207].

However, cell replacement therapy faces even more profound translational challenges that must be acknowledged. The risk of tumor formation, particularly from undifferentiated pluripotent stem cells, represents a significant safety concern that requires rigorous quality control measures and complete differentiation before transplantation [208]. The survival rate of transplanted cells is typically low, with the vast majority dying within days to weeks after engraftment due to the hostile microenvironment of the diseased brain, which is characterized by inflammation, oxidative stress, and reduced trophic support [209, 210]. Even when cells survive, achieving functional synaptic integration into existing neural circuits is extremely challenging; transplanted neurons must form appropriate afferent and efferent connections, which are rarely demonstrated convincingly [211]. The possibility of host immune rejection, even with autologous iPSC‐derived cells, exists and may require immunosuppression, with its attendant risks [212]. The scalability and standardization of cell production under good manufacturing practice (GMP) conditions are technically demanding and prohibitively expensive for many research groups. Moreover, for cholinergic cell replacement to be effective in diseases with widespread cortical pathology, the transplanted cells would need to establish extensive projections over long distances, which is unlikely with focal transplantation [213, 214]. The ethical considerations surrounding the use of embryonic stem cells, though partially addressed by iPSC technology, remain a concern in some regions [215]. Most importantly, robust evidence for clinically meaningful cognitive improvement in human patients is still lacking, and it remains unclear whether cell transplantation can modify disease progression beyond providing transient trophic effects [216, 217].

Although gene and cell therapies show great potential in restoring cholinergic function, they remain largely at the preclinical or early‐phase clinical trial stage, with numerous fundamental hurdles to overcome before they can be considered viable clinical options for neurodegenerative diseases. Key challenges requiring urgent attention include: improving the safety and specificity of gene delivery vectors; developing non‐invasive or minimally invasive delivery methods that ensure adequate biodistribution; enhancing the survival, differentiation, and functional integration of transplanted cells; and establishing rigorous preclinical models that accurately predict human responses [218, 219]. In addition, the high cost and complexity of these advanced therapy medicinal products will require healthcare systems to develop new reimbursement and delivery models if they are ever to reach routine clinical use. With continuous advances in vector engineering, differentiation protocols, and cell manufacturing, these approaches may eventually contribute to disease modification, but this is likely to be a long‐term, incremental process rather than an imminent clinical reality. Realistic expectations, rigorous safety monitoring, and stepwise clinical development will be essential to translate the therapeutic promise of gene and cell‐based strategies into tangible benefits for patients [218].

## 6 Conclusion and Future Perspectives

Current evidence supports a strategic evolution in cholinergic therapies for neurodegenerative diseases, moving beyond the symptomatic cholinergic enhancement of acetylcholinesterase inhibitors toward more precise and integrative approaches. However, translating this potential into clinical reality requires grounding future directions in achievable, evidence‐driven steps rather than broad, long‐term agendas.

### 6.1 Precision Pharmacology: Advancing Receptor‐Specific and Multi‐Target Strategies

While AChEIs will remain a mainstay of symptomatic treatment, the field must prioritize the clinical validation of receptor subtype‐selective modulators and multi‐target directed ligands. A critical near‐term task is the continued development and rigorous Phase III testing of M1 positive allosteric modulators (PAMs), such as TAK‐071, to confirm their cognitive benefits and establish their safety profile in larger, well‐controlled trials [220]. Concurrently, the therapeutic potential of dual AChE/BChE inhibitors like rivastigmine should be further explored, particularly in late‐stage patients where BChE activity is upregulated. For emerging approaches like protein degradation (PROTACs), the immediate priority is to address fundamental barriers to central nervous system application, including blood–brain barrier permeability and target specificity in vivo [221, 222]. Progress in these areas will determine whether they can realistically contribute to medium‐term treatment goals.

### 6.2 Circuit Modulation: Refining and Combining Neuromodulation Protocols

Neuromodulation techniques offer a complementary pathway by directly engaging dysfunctional neural circuits. The most promising near‐term objective is to optimize and standardize stimulation parameters for existing non‐invasive techniques—transcutaneous vagus nerve stimulation (tVNS), repetitive transcranial magnetic stimulation (rTMS), and transcranial direct current stimulation (tDCS)—through well‐powered, sham‐controlled trials that define optimal dosing, frequency, and target engagement. For invasive approaches like deep brain stimulation (DBS) of the nucleus basalis of Meynert (NBM) and pedunculopontine nucleus (PPN), the immediate focus should be on identifying patient subgroups most likely to benefit, using baseline structural and functional imaging to guide patient selection [223]. A key next step is to systematically investigate the additive or synergistic effects of combining neuromodulation with pharmacological agents. For example, pairing tVNS or tDCS with cognitive training, or combining NBM‐DBS with a cholinergic drug, represents a realistic and testable strategy to enhance overall efficacy without relying on unproven closed‐loop systems.

### 6.3 Imaging and Biomarkers: Translating Visualization Into Clinical Decision‐Making

Advanced imaging techniques like VAChT‐PET provide invaluable insights into cholinergic integrity, but their immediate clinical utility lies in informing treatment stratification, not in complex closed‐loop control. The most actionable path forward is to use baseline cholinergic imaging (e.g., VAChT‐PET or structural MRI of the basal forebrain) to identify patients with preserved cholinergic terminals who are more likely to respond to AChEIs or NBM‐DBS, thereby avoiding ineffective treatments and enabling more efficient trial designs [175]. Similarly, short‐latency afferent inhibition (SAI), a transcranial magnetic stimulation‐based electrophysiological marker of cholinergic function, holds promise as a bedside tool for monitoring treatment response in individual patients [224]. Establishing the predictive value of these biomarkers in prospective clinical trials is a concrete, high‐priority goal that precedes any vision of real‐time, imaging‐driven closed‐loop systems.

In summary, therapeutic exploration is advancing along a trajectory from purely chemical enhancement toward integrated circuit‐level intervention. However, the most effective strategy for the coming years is not to seek a single magic bullet, but to pursue a pragmatic, multi‐pronged approach. This involves: (i) rigorously validating next‐generation pharmacological agents in targeted patient populations, (ii) optimizing and personalizing neuromodulation protocols based on simple, accessible biomarkers, and (iii) designing rational combination trials that leverage the complementary mechanisms of drugs and stimulation. By focusing on these achievable steps, the cholinergic system will remain a central and productive guide in our efforts to meaningfully improve outcomes for patients with neurodegenerative diseases.

## Author Contributions

Jian‐jun Yang and Qingsheng Meng conceived the idea for the article. Lei Lei and Xiaoyu Hu performed the literature search and data analysis. Lei Lei drafted the manuscript, and all authors (Ruida Lu, Xiaoyu Hu, Jian‐jun Yang, Qingsheng Meng) participated in its critical revision. All authors approved the final version for publication.

## Funding

This work was supported by grants from the National Natural Science Foundation of China (U23A20421 to J.‐j.Y.); Scientific Research and Innovation Team of The First Affiliated Hospital of Zhengzhou University (ZYCXTD2023012 to J.‐j.Y.).

## Conflicts of Interest

The authors declare no conflicts of interest.

## Data Availability

Data sharing is not applicable to this article, as no datasets were generated or analyzed during the current study.
